# Three-Dimensional Phase-Sensitive Inversion-Recovery Turbo FLASH Sequence for the Assessment of Left Ventricular Myocardial Scar in Swine

**DOI:** 10.1371/journal.pone.0078305

**Published:** 2013-10-23

**Authors:** Xiuyu Chen, Minjie Lu, Gang Yin, Tao Zhao, Xiaoning Shao, Ranxu Zhao, Yue Tang, Jing An, Shiliang Jiang, Shihua Zhao

**Affiliations:** 1 Department of Radiology, State Key Laboratory of Cardiovascular Disease, Fuwai Hospital, National Center for Cardiovascular Diseases, Chinese Academy of Medical Sciences and Peking Union Medical College, Beijing, China; 2 Dapartment of Magnetic Resonance Imaging, the First Affiliated Hospital of Zhengzhou University, Zhengzhou, Henan Province, China; 3 Department of Pathology, State Key Laboratory of Cardiovascular Disease, Fuwai Hospital, National Center for Cardiovascular Diseases, Chinese Academy of Medical Sciences and Peking Union Medical College, Beijing, China; 4 Animal Experimental Center, State Key Laboratory of Cardiovascular Disease, Fuwai Hospital, National Center for Cardiovascular Diseases, Chinese Academy of Medical Sciences and Peking Union Medical College, Beijing, China; 5 APPL department, Siemens Shenzhen Magnetic Resonance Ltd, Shenzhen, China; University of Washington School of Medicine, United States of America

## Abstract

**Objectives:**

We sought to evaluate the feasibility and accuracy of free-breathing three-dimensional (3D) phase-sensitive inversion-recovery (PSIR) Turbo FLASH sequence for noninvasive assessment of left ventricular myocardial scar in swine models.

**Materials and Methods:**

Nine Chinese minipigs with experimentally induced acute myocardial infarction were studied. At 1 week and the study endpoint 4 weeks after myocardial infarction surgery, the 3D and 2D contrasted cardiac magnetic resonance (CMR) imaging were performed randomly by using a 1.5T clinical MR imaging system. Comparisons of myocardial scar volume (in cubic centimeters), scar transmurality (on a 5 points scale) and image quality (on a 4 points Likert scale) were performed by using the Pearson correlation and Bland-Altman analysis (for myocardial scar volume) or κ statistics (for transmurality) or Wilcoxon signed rank test (for image quality).

**Results:**

In 6 of the 9 pigs, all procedures were successfully completed. In these pigs, a total of 48 segments with myocardial scars were detected by both 3D and 2D sequences, and there was good agreement for classification of scar transmurality (κ=0.930). The scar volume determined by triphenyltetrazolium chloride (TTC) staining (3.52±1.40cm^3^) showed a good correlation with both 3D (3.54±1.36cm^3^, r=0.957, *P*=0.003) and 2D sequence (3.53±1.26cm^3^, r=0.942, *P*=0.005) at 4 weeks. And there were good correlation between scar volumes obtained from 3D and 2D techniques (r=0.859, *P*<0.001) at both time points. Both 3D and 2D images detected a small reduction of scar volume from week 1 to week 4 by a factor of 1.179 and 1.176, respectively. Although slightly more artifacts were observed on 2D PSIR images, the overall image quality was not significantly different between the two sequences (3.17±0.83 for 2D vs. 3.25±0.75 for 3D, P =0.655).

**Conclusions:**

The free-breathing 3D PSIR Turbo FLASH sequence enables accurate assessment of left ventricular myocardial scar.

## Introduction

Late gadolinium enhancement (LGE) cardiac magnetic resonance (CMR) is an established method for the detection and characterization of left ventricular myocardial scar [1,2]. The degree of infarct transmurality has been proved to be a critical predictor for restoration of regional contractile function after revascularization and prognosis [3].

The current reference standard for LGE-CMR is two-dimensional (2D) segmented inversion-recovery rapid spoiled gradient echo (Turbo FLASH in Siemens) sequence[4]. However, it requires consistent and repeated breath holds. Only one slice is acquired per breath hold, thus, 10-16 breath holds each lasting at least 10 heartbeats are necessary to cover the entire left ventricle on short-axis orientation [5]. Furthermore, this approach may suffer from the partial volume effects owing to the relatively large slice thickness [6]. 

In previous studies, three-dimensional (3D) inversion-recovery (IR) balanced steady-state free precession (b-SSFP) sequence or single shot IR Turbo FLASH [7-11] was used to assess the entire left ventricle within one breath hold. However, the spatial resolution is suboptimal and the voxel is not isotropic, so that reformation to arbitrary views from the image data is restricted for the visualization of myocardial scar. On the other hand, in navigator-gated 3D sequence [12,13], the inversion time (TI) to null healthy myocardial signal may significantly change over the long acquisition time, as TI is dependent on the clearance of contrast agent across the scan duration [12]. The phase-sensitive inversion-recovery (PSIR) technique used in this 3D study eliminates the necessity to precisely choose the optimal TI and allows consistent contrast between normal and inactive myocardium during the whole acquisition [14]. Furthermore, it can obtain a thinner slice thickness and isotropic data set for reformatting in all directions during a relatively long acquisition time [14,15].

The 3D PSIR Turbo FLASH sequence has been published earlier that it is a promising technique for assessment of myocardial scar in patients [4,16,17]. However, the accuracy has not been pathologically validated. Accordingly, the purpose of this study was to evaluate the feasibility and accuracy of a free-breathing 3D PSIR Turbo FLASH sequence for noninvasive assessment of left ventricular myocardial scar and compare it with the clinically established breath-hold 2D PSIR turbo FLASH sequence in swine models.

## Materials and Methods

### Animal Model

Nine Chinese mini-pigs (27±3kg, 12.0±2.0months) were used in this study. Animals were sedated with ketamine (35mg/kg), induced with valium (1.5mg/kg); then endotracheally intubated and mechanically ventilated with a Narkomed ventilator. Anesthesia was maintained through the intravascular injection of ketamine and valium. A midline sternotomy was performed and the heart was suspended by a pericardial cradle. The distal left anterior descending artery beyond the first diagonal branch was dissected free which was then permanently ligated to produce myocardial infarction (MI). 

### Ethics statement

All animal studies were approved by Ethics Committee for Animal Study in Fuwai hospital (approved number 2011-438) and complied with the “Guide for the Care and Use of Laboratory Animals” published by the US National Institutes of Health. All the animals received postoperative antimicrobial therapy (cephazoline 1.0g, intramuscular injection, twice daily for 3d) and buprenorphine (0.3mg, twice daily for 3 d) for postoperative pain.

### TTC staining

At the study endpoint (after the second CMR examination, 4 weeks post-MI), the animals were euthanized and the hearts were excised which were then stiffened by immersion in 95% ethanol, precooled to -80°C, and sectioned from base to apex into 6-mm-thick slices with a professional slicer. All slices were then incubated in 1% triphenyltetrazolium chloride (TTC) for 30 minutes at 37°C and digitally photographed, and the volumes of infarction (in cubic centimeters) were determined by using image analysis software (ImageJ; National Institutes of Health), by two observers (TZ and GY) by consensus.

### CMR Protocol

All examinations were performed on a clinical 1.5T MR scanner (MAGNETOM Avanto^®^, Siemens^®^ Healthcare, Erlangen, Germany) with a high-performance gradient system (45mT/m; maximum slew rate, 200 mT/m/ms), and 12-channel surface phased array coils. Wireless MR-compatible ECG was used for MR gating. Each of the pigs underwent MRI at 1 week and 4 weeks post-MI. All animals were anesthetized prior to MR examination with administration of sodium pentobarbital (25mg/kg). Transversal, sagittal and coronary scout images were obtained firstly. Arbitrarily angulated scout images were then acquired. Retrospective electrocardiographic gating cine imaging was performed using a segmented balanced steady-state free precession (b-SSFP) sequence in the 3 long-axis views (2-chamber, 4-chamber and 3-chamber) and continuous short axis views covering the whole left ventricle from base to apex. 

For evaluation of myocardial viability, a clinically established 2D PSIR Turbo FLASH sequence was used as the reference standard for the 3D PSIR Turbo FLASH sequence. A stack of 2D short-axis images without gap and 3D images covering the entire left ventricle in a transversal slab were acquired in random order. All images were acquired during free-breathing at 10-15 minutes after the contrast administration (Gadopentetate dimeglumine, Magnevist, Bayer Schering Pharma AG, Germany. 0.2mmol/kg). 

Typical imaging parameters for 2D PSIR Turbo FLASH sequence were: repetition time/echo time (TR/TE)=8.4/3.25ms, flip angle=25°, bandwidth=140Hz/pixel, number of k-space lines per cardiac cycle=25 (filled the k-space sequentially), data window duration=167ms, field of view (FOV) =340×270mm, matrix=156×256, pixel size=1.8×1.3mm, slice thickness=6mm, slice gap=0mm. Generalized autocalibrating partially parallel acquisition (GRAPPA) was used to accelerate the data acquisition (factor=2). Due to the PSIR technique, a nominal value of TI =300ms was used, rather than an additional breathhold TI scout technique was performed to determine the optimal TI to suppress the normal myocardium [14,15]. The sequence mentioned above was previously reported [18,19].

For the 3D PSIR Turbo FLASH sequence, a crossed pair navigator pulse was used for respiratory gating. After a nonselective IR pulse, a selective IR pulse was applied to restore the magnetization of the navigator beam for subsequent navigator echo acquisition [20]. A fixed TI value of 300ms was used. IR prepared image data was acquired using Turbo FLASH readout. Every other heartbeat, a proton weighted phase map with low flip angle (5°) was acquired and used to perform a surface coil correction and obtain a reference phase within the scope of the PSIR reconstruction [14]. Typical imaging parameters were: TR/TE=3.7/1.83ms, flip angle=25°, TI=300ms, bandwidth=504Hz/pixel, number of k-space lines per cardiac cycle=35 (filled the k-space sequentially), data window duration=129ms, FOV=300×260mm, matrix=140×160, slices per slab=42±6, slabs=1, voxel size=2.0×2.0×2.0mm, GRAPPA factor =2. The acceptable window of navigation was 4 mm, and respiratory motion adaptation (slice tracking) was used. A noise reduction technique was applied to the 3D sequence, which resulted in suppression of noise in areas without detectable MR signal.

### Image Reconstruction and Analysis

Before analysis, all 3D raw data were transferred to an offline workstation (Argus®, Siemens), and then constructed into a contiguous set of short-axis views matching the view orientation and slice thickness of 2D images. As a result, three 2-mm slices without a gap were reconstructed to match the coverage of one 2D slice.

The image quality of 2D and 3D images was visually evaluated and scored based on a 4 points Likert scale (1 = poor, non diagnostic; 2 = fair, diagnostic maybe impaired; 3 = good, some artifacts but not interfering in diagnostics, 4 = excellent, no artifacts), and results were expressed as a mean score per subject. All images were evaluated by two observers (GY and XS) by consensus. 

The volume (in cubic centimeters) of hyperenhanced myocardium (infarcted myocardium) was measured on a dedicated evaluation workstation (Leonardo; Siemens Medical Solutions, Erlangen, Germany) by the same observers (GY and XS) by consensus. LGE was considered present in areas with signal intensity exceeding > 2SD that of normal myocardium signal in the same section[21]. The scar volume was calculated by multiplying the sum of all scar areas of each slice with slice thickness plus slice gap (for both 2D and 3D: slice gap=0). .

As recommended, a previously described 30-segment model was used for the animal study[1,9] to visually evaluate the transmural extent of scar on 2D and 3D images. Two observers (TZ and GY) scored the transmural extent of scar in each segment by consensus, according to a five-point grading system [22](0=no infarction, 1=transmural extent of infarction 1–25% of left-ventricular wall thickness, 2=26-50%, 3=51-75%, 4=76-100%).

### Statistical Analysis

All data are expressed as means±S.D. Pearson correlation and linear regression analysis were used to analyze the correlation between the scar volume measured from MR imaging and those measured from TTC staining. Results were conﬁrmed by Bland-Altman analysis (results of which are expressed as means±1.96· SD = means ± limits of agreement). A paired Student’s *t* test was used to compare the scar volumes obtained from 2D and 3D images. Wilcoxon signed rank test was used to compare overall image quality scores between the sequences. Agreement on the transmural extent of the scar between the 3D and 2D MR imaging sequence was evaluated using the Kappa test. All statistical analyses were conducted by using statistical software package SPSS 16.0 (SPSS, Chicago, IL). A value of *P* < 0.05 was considered significant.

## Results

**Figure 1:**
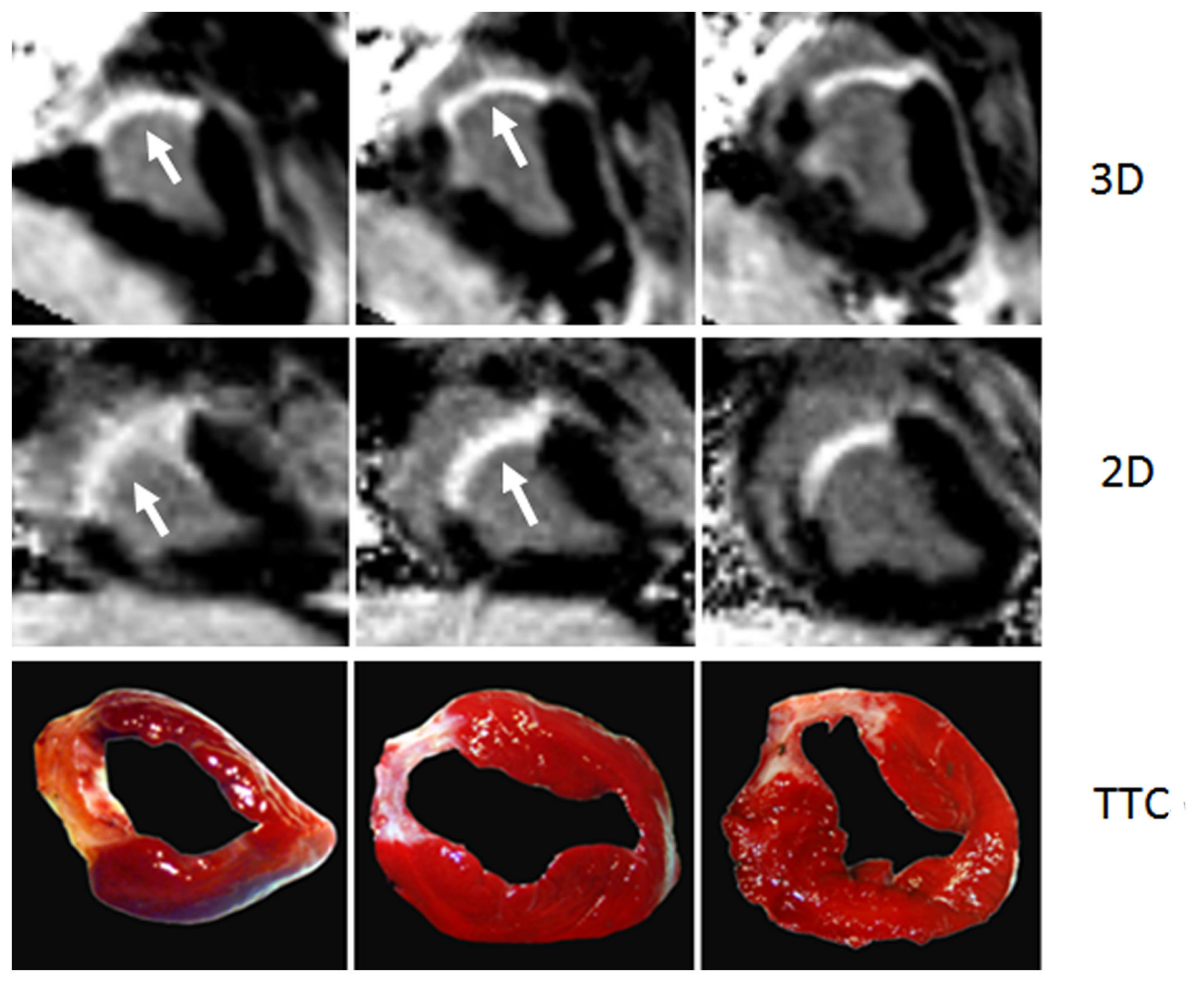
Comparison of short-axis MR images with TTC staining. Example of 3D, 2D images and TTC staining obtained from the same pig 4 weeks post MI. It showed good agreement in depiction of the extent of myocardial scar, and the borders of hyperenhanced regions were much clearer on 3D images (arrows).

One pig did not survive the infarct procedure and two died on the ﬁrst day after MI induction. In the remaining 6 pigs, all procedures including MR examinations and TTC staining were successfully completed (Figure 1). The average acquisition time for 3D sequence was 5.1±1.3 min, with an average navigator efficiency of 51%, while the 2D scan took 2.1±0.6 min to collect 10±3 slices across the whole LV. Although slightly more artifacts were observed on 2D images, the overall image quality was not significantly different between the two sequences (3.17±0.83 for 2D vs. 3.25±0.75 for 3D, P = 0.655). 

In these 6 pigs, a total of 48 segments with LGE were observed on both sequences. No hyperenhanced LV segment on the 2D images was missed on the 3D images. For classification of transmural extent of myocardial scar of the 180 segments based on the five-point grading system, these two sequences were in good agreement (κ = 0.930). 

**Figure 2:**
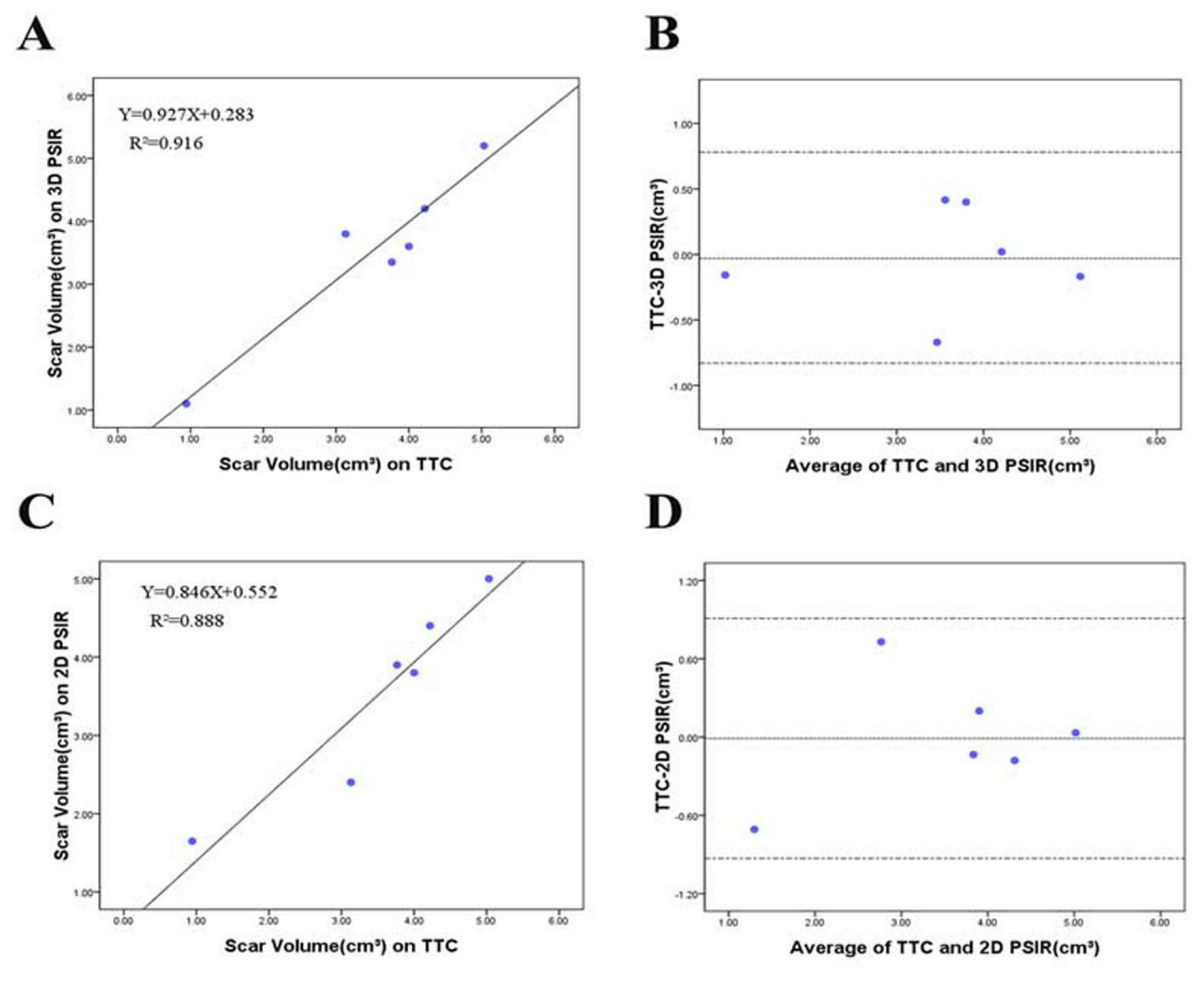
Relationship between scar volumes on MR images and TTC staining. The relation between MR images and TTC staining for scar volume was evaluated for 6 animals at 4 weeks. (A and B) 3D sequence and TTC staining showed a good correlation for scar volume (r=0.957, *P*=0.003). (C and D) 2D sequence and TTC staining showed a good correlation for Scar volume (r=0.942, *P*=0.005).

**Figure 3:**
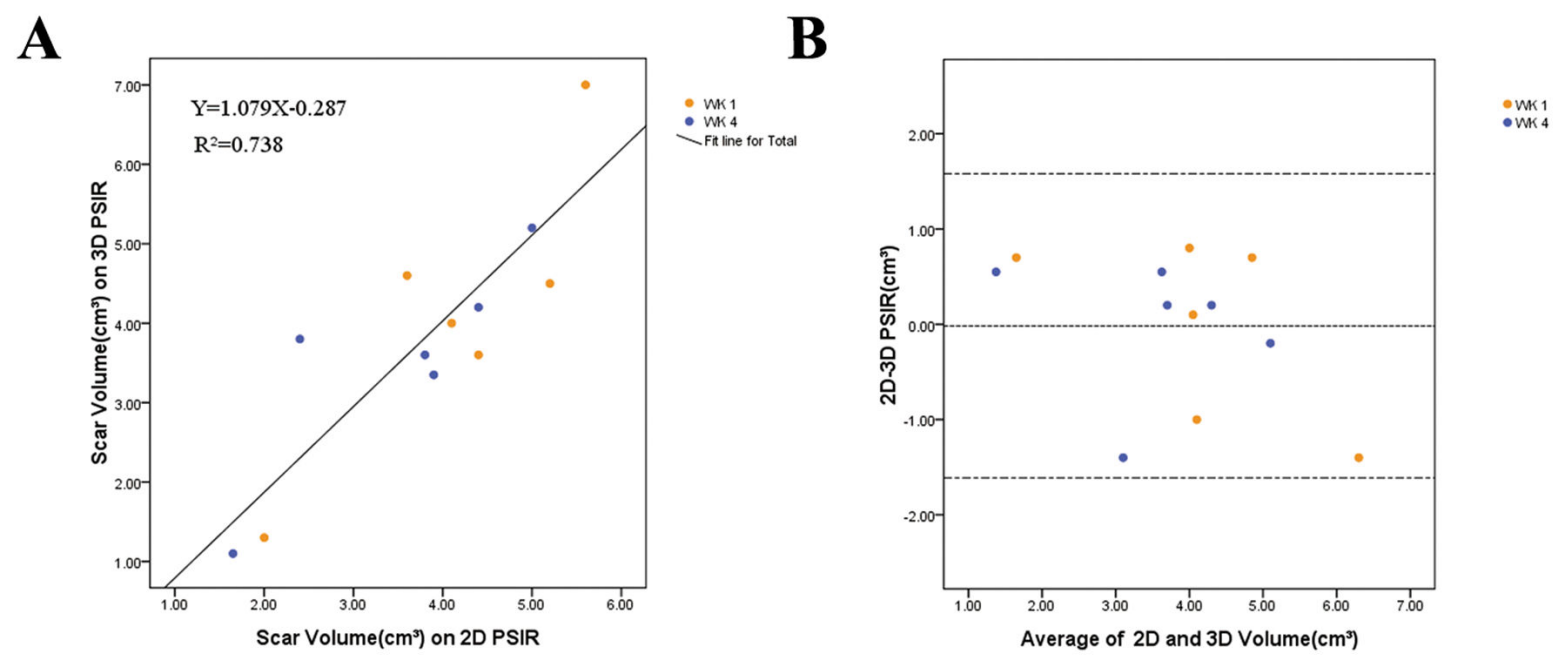
Relationship between scar volume on 3D and 2D images. The relation between 3D and 2D images for scar volume was evaluated for 6 animals at both time points. (A) Linear regression plot shows a good correlation between scar volume on 3D PSIR images and 2D PSIR images (r=0.859, *P*<0.001). (B) Bland-Altman analysis showed there was no systematic under- or overestimation of scar volume with 3D PSIR images, and limits of agreement were sufficiently small compared with average scar volume.

**Figure 4:**
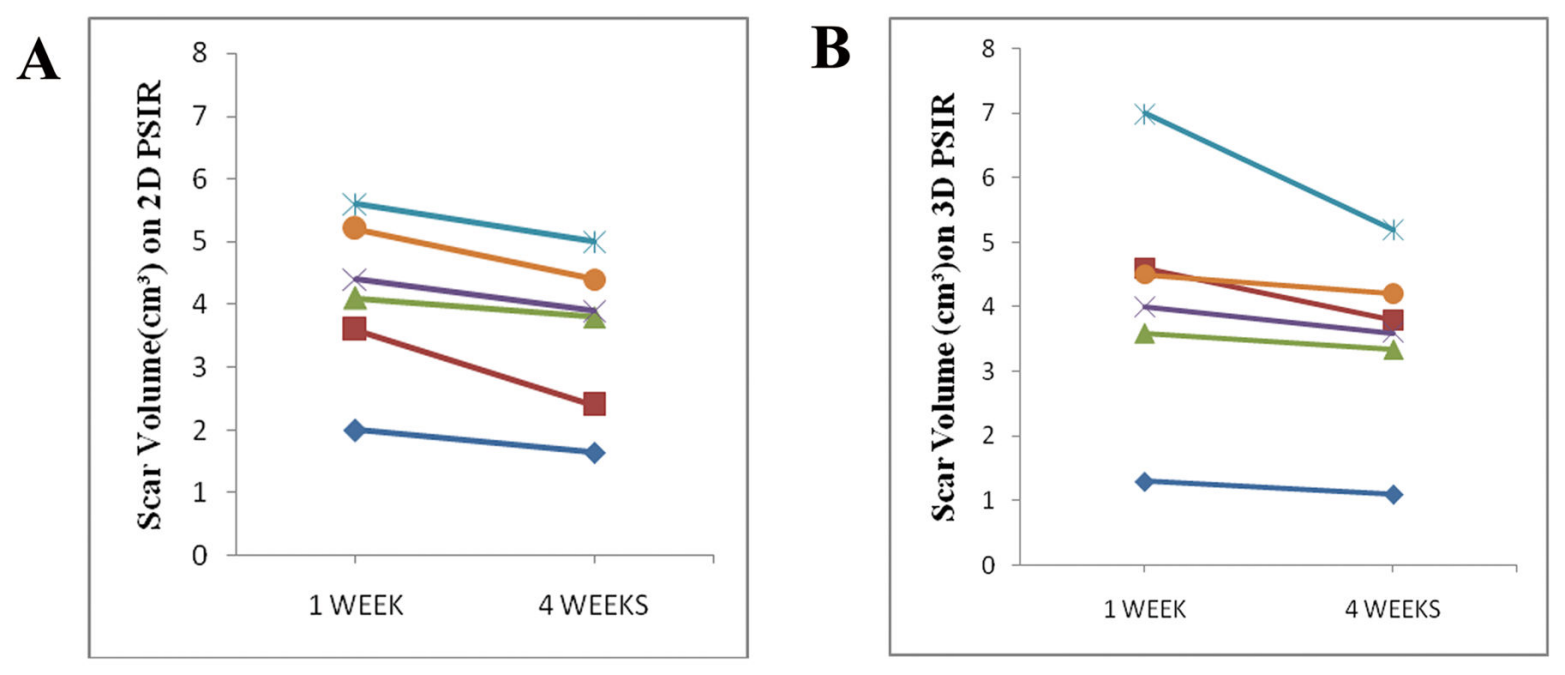
Comparison of results at 1 week and 4 weeks. Results of scar volume changing between 1 week and 4 weeks on 2D (A) and 3D (B) images, respectively, which shows an reduction trend in infarct volume from week 1 to week 4.

The scar volume determined by TTC staining (3.52±1.40cm^3^) showed a good correlation with both 3D (3.54±1.36cm^3^, r=0.957, *P*=0.003) and 2D sequence (3.53±1.26 cm^3^, r=0.942, *P*=0.005) at 4 weeks. Bland-Altman analysis showed that the limits of agreement (mean±1.96·SD) of TTC staining compared with that of 3D PSIR images were -0.03±0.80 cm^3^ and compared with that of 2D PSIR images were -0.01±0.92 cm^3^(Figure 2). At both time points there were good correlation between scar volumes obtained from 3D and 2D techniques (r=0.859, *P*<0.001). Bland-Altman analysis showed that the limits of agreement (mean ± 1.96·SD) of 3D images compared with that of 2D images were -0.02 ± 1.59cm^3^ (Figure 3). In addition, this study demonstrated a small reduction of scar volume from 1 week to 4 weeks both in 3D and 2D technique by a factor of 1.179 and 1.176, respectively (Table 1, Figure 4).

**Table 1:** Comparison of scar volume obtained from 3D and 2D images at the two time points.

Parameters	3D	2D	*P* value
Total(n=12)	3.85 ± 1.57	3.84 ± 1.25	0.945
Week 1(n=6)	4.17 ± 1.84	4.15 ± 1.28	0.968
Week 4(n=6)	3.54 ± 1.36	3.53 ± 1.26	0.958
Changes(n=6)	0.63 ± 0.61	0.63 ± 0.33	0.975

Note.―Values are expressed as mean ± SD

## Discussion

To our knowledge, this is the first study to use the free-breathing 3D PSIR Turbo FLASH sequence in swine models for the evaluation of LV myocardial infarction. Our results suggested that the 3D PSIR sequence was reliable and feasible for assessment of LV myocardial scar without the need of breath holds. This study provided significant pathological evidences for previous human studies [4,12,17] and introduced the 3D PSIR sequence as an alternative LGE-CMR technique particularly for patients who were unable to comply with breath-holding requirements. 

In this study, the scar volumes measured from the free-breathing 3D PSIR images were highly correlated and in good agreement with those measured from 2D PSIR images and TTC staining, and we observed similar scar volume between 3D and 2D sequence in both time points. These findings were supported by Nguyen TD’s [12] and Peters DC’s [23] study, which described that the scar volumes measured by the 2D and 3D LGE methods were in narrow limits of agreement or close agreement. Besides, according to an animal research by Kim RJ et al [6], the size and shape of areas exhibiting LGE were identical to areas suffering irreversible injury confirmed by TTC staining. However, the studies by Kino A [4] and Gang Y [17] showed the 3D sequence detected larger scar volume than 2D sequence, which was probably due to the discontiguous scan in the 2D sequence in these studies. In contrast, the 2D short-axis images were acquired without gap in this animal study.

LGE-CMR for noninvasive assessment of myocardial viability was an established approach to clearly distinguish the transmural extent of acute myocardium necrosis and scar. The 2D PSIR Turbo FLASH was used as a standard reference in this study because it was previously demonstrated [8,22]and widely used to assess myocardial viability in routine clinical practice [24-28]. However, this technique requires good patient cooperation. 10-16 breath holds each lasting at least 10 heartbeats are necessary to assess the entire left ventricle [6]. On the other hand, this approach has the potential for missing small or atypical lesions at slice interfaces and inaccuracy of myocardial scar quantification owing to noncontiguous slice acquisition [3].

 3D LGE imaging, covering the entire heart in one single acquisition, has some attractive advantages, including high signal-to-noise ratios and thinner, contiguous sections, allowing multiplanar reconstruction in any desirable axis [4,16]. However, the major challenge for previous 3D approaches without PSIR technique is the long imaging time, during which the optimal TI to null healthy myocardial signal may significantly change, so that the suboptimal suppression of viable myocardium may occur when TI is fixed [29,30]. 

In this study, PSIR technique was applied to 3D Turbo FLASH sequence in order to reduce the effects of dynamic changes in TI during the long scan, contributing to a consistent image contrast between normal and infarct myocardium. In a previous study that used a 3D inversion-recovery fast low-angle shot sequence, only moderate agreement for scar transmurality was observed probably owing to the rather long acquisition time of 293 msec per heartbeat [8]. In contrast, in this study, the imaging time per heartbeat for the 3D PSIR Turbo FLASH sequence was 129 msec. Another 3D technique called 3D inversion-recovery (IR) balanced steady-state free precession (b-SSFP) sequence was used in a clinical and animal study, which showed good agreement with the corresponding 2D sequence and decreased the acquisition time by a factor of nine to 159 msec [6]. However, this approach still requires breath hold which would be a disadvantage in patients who have difficulty holding their breath. 

Compared with human, the diaphragmatic movement of the pig was relatively week, and the quality of 2D images acquired during free-breathing was quite acceptable. Although there were still slightly more artifacts on 2D images, the image quality between 3D and 2D images was comparable in this study. However, according to our clinical practice [17], 2D images would suffer from obvious respiratory artifacts if patients could not repeat breath-holds. In contrast, the 3D approach eliminated the necessity to hold breath during the scan in which navigator echoes were obtained to synchronize respiratory, without compromising image quality. This could be important clinically because many patients with coronary artery disease, in whom viability imaging with CMR would be relevant, cannot perform consistent and repeated breath holds required for the 2D techniques. 

Finally, in the present study, both 3D and 2D sequence observed a small reduction of scar volume from 1 week to 4 weeks by a factor of 1.179 and 1.176, respectively, and this could be explained by infarct shrinkage during the transition from myocyte necrosis to collagenous scar [6,31].

## Study Limitations

Our study had some limitations. First, all MR imaging was conducted without any medication such as beta-blocker, although the animals had elevated heart rates above 80 to 100 beats/min, which probably reduced the overall image quality. Second, the signal-to-noise ratio of 2D PSIR and 3D PSIR images was not measured because the noise level was modified on the phase sensitive images and the noise-only regions were no longer valid for estimating the background noise[14]. Finally, here we only had animal but no human studies, because our aim was to evaluate the accuracy of this 3D PSIR sequence for assessment of myocardial scar by a true gold standard.

## Conclusion

This animal study suggests that the free-breathing 3D PSIR Turbo FLASH sequence is feasible for accurate assessment of left ventricular myocardial scar volume and transmurality. This finding provides significant pathological evidences for previous human studies and introduces the 3D PSIR sequence as an alternative LGE-MR technique particularly for patients who were unable to comply with breath-holding requirements.
