# Correction: Three-Dimensional Phase-Sensitive Inversion-Recovery Turbo FLASH Sequence for the Assessment of Left Ventricular Myocardial Scar in Swine

**DOI:** 10.1371/annotation/66ee2369-2bcb-4af0-be75-7212c5463241

**Published:** 2013-11-19

**Authors:** Xiuyu Chen, Minjie Lu, Gang Yin, Tao Zhao, Xiaoning Shao, Ranxu Zhao, Yue Tang, Jing An, Shiliang Jiang, Shihua Zhao

The first and second authors, Xiuyu Chen and Minjie Lu, should be indicated as having contributed equally to the work.

In addition multiple funding organizations and grants were incorrectly omitted from the Funding Statement. The Funding Statement should read: "This study was supported in part by the Research Grant of National Natural Science Foundation of China (81000604 and 81370036 )，PUMC Youth Fund and the Fundamental Research Funds for the Central Universities (2009-xhj04# and 3332013105), and Grant for Talent Research Star of Fuwai Hospital (2012‐FWXX01). The funders had no role in study design, data collection and analysis, decision to publish, or preparation of the manuscript."

